# News media coverage of extreme risk protection order policies surrounding the Parkland shooting: a mixed-methods analysis

**DOI:** 10.1186/s12889-021-11909-z

**Published:** 2021-11-02

**Authors:** Rocco Pallin, Amanda J. Aubel, Christopher E. Knoepke, Veronica A. Pear, Garen J. Wintemute, Nicole Kravitz-Wirtz

**Affiliations:** 1 Violence Prevention Research Program, Department of Emergency Medicine, University of California Davis School of Medicine, 2315 Stockton Blvd, Sacramento, CA 95817 USA; 2 California Firearm Violence Research Center at UC Davis, 2315 Stockton Blvd, Sacramento, CA 95817 USA; 3 Division of Cardiology, University of Colorado School of Medicine, 13199 East Montview Boulevard, Suite 300, Aurora, CO 80045 USA; 4 Adult and Child Consortium for Outcomes Research and Delivery Science, University of Colorado School of Medicine, 13199 East Montview Boulevard, Suite 300, Aurora, CO 80045 USA

**Keywords:** Firearms, Media coverage, News media, United States, Injury prevention, Firearm policy, ERPO, Extreme risk protection order, Extreme risk law, Mass shooting, Parkland shooting, Red flag law, Media reporting

## Abstract

**Background:**

Following the 2018 mass shooting at Marjory Stoneman Douglas High School in Parkland, Florida, there was a dramatic increase in media coverage of extreme risk protection orders (ERPOs) and in state policy proposals for ERPO laws. This study documents the frequency of news coverage of ERPOs throughout 2018 and examines the narratives used by media outlets to describe this risk-based firearm policy.

**Methods:**

Using a mixed-method descriptive design, we examine the frequency of national news media coverage of ERPO legislation in 2018, before and after the Parkland shooting, and analyze the content of news articles related to a sample of states that considered ERPO legislation after the shooting.

**Results:**

We find a sharp increase in the frequency of articles related to ERPOs following the Parkland shooting and smaller increases in coverage surrounding ERPO policy proposals and other public mass shootings that year. Nearly three-quarters of articles in our content analysis mentioned the Parkland shooting. The news media often mentioned or quoted politicians compared to other stakeholders, infrequently specified uses for ERPOs (e.g., prevention of mass violence, suicide, or other violence), and rarely included evidence on effectiveness of such policies. More than one-quarter of articles mentioned a mass shooting perpetrator by name, and one-third of articles used the term “gun control.”

**Conclusions:**

This study describes the emerging public discourse, as informed by media messaging and framing, on ERPOs as states continue to debate and implement these risk-based firearm violence prevention policies.

**Supplementary Information:**

The online version contains supplementary material available at 10.1186/s12889-021-11909-z.

## Background

Firearm violence persists as a significant and distinctly American problem, with more than 39,000 deaths each year [1]. Although they are not a major cause of gun deaths, public mass shootings draw immense attention and news media coverage. For many, these tragedies bring firearm violence into acute focus, stimulating public discourse about its causes and consequences and possible strategies for preventive action. Similarly, when attention to firearm violence is heightened following a high-profile mass shooting, policymakers and public figures often find increased opportunities and impetus to speak on legislative strategies for addressing gun violence. Policy advocates may also find themselves in a unique spotlight that amplifies their messaging, whether in favor of or opposed to gun policy changes.

This was exemplified after the February 2018 mass shooting at Marjory Stoneman Douglas High School in Parkland, FL (hereafter, “Parkland”), where seventeen students and teachers were killed and seventeen more were injured. In particular, Parkland precipitated intense nationwide discussion about extreme risk protection order (ERPO) laws and states’ rapid adoption of these laws. ERPO laws are sometimes colloquially referred to as “red flag” laws. They are targeted, flexible, and proactive tools for temporarily removing access to firearms from an individual whom a judge has deemed to be at increased risk of firearm-related harm to self or others and who is not otherwise prohibited from firearm ownership.

Many violence prevention advocates, policymakers, and practitioners believed Parkland epitomized the need for ERPO legislation. Prior to the attack, the shooter made repeated threats of violence, including specific threats against schools, and had come to the attention of both the county sheriff’s department and the Federal Bureau of Investigation [2, 3]. However, the shooter did not have prior criminal justice system involvement that prohibited him from legally purchasing or possessing firearms. In addition, reports suggest that mental health professionals did not believe he met the criteria for emergency mental health evaluation and treatment, which could result in a short-term firearm prohibition [4]. Experts have since argued that an ERPO law—which offers an additional mechanism for concerned law enforcement, family, and others to reduce an at-risk person’s access to firearms—might have helped to prevent the Parkland shooting [5, 6].

As a primary source of information about pressing social and health problems like gun violence, the news media both reflects and shapes public discourse about these issues and potential policy solutions [7–9]. Past research has investigated news narratives about gun violence and gun policy, including frameworks such as “gun control” (vs “gun rights”) and “dangerous people” (vs “dangerous weapons”), in the aftermath of high-profile mass shootings [10–12]. However, this is the first study, to our knowledge, to examine coverage of ERPO laws during the period surrounding Parkland, after which uptake of the policy increased dramatically, from three states (and two with analogous “risk warrant” laws) at the outset of 2018 to 27 states considering or having considered ERPO legislation by the end of the year.

To assess the salience of public discourse on ERPOs in the context of Parkland, we first analyzed the frequency of national news media coverage of ERPO policies before and after the shooting. Then, to examine media framing of ERPOs, we explored the content of a sample of news articles about six states that first considered ERPO legislation following Parkland. Our findings summarize messaging about ERPOs to inform broader discussions about how the news media presents information and may shape public understandings of key social and health problems and corresponding policy interventions after precipitating, high-profile incidents.

## Data and methods

### Study design and data collection

We used a mixed-method descriptive design consisting of frequency analysis and content analysis informed by our previous work [13], as well as by previous research on news coverage of gun violence [14]. To assess news media coverage, we collected news articles from Nexis Uni and Newsbank (“Accesss World News” database), two of the largest, web-based full-text databases for current news. We constrained our searches to print news media (i.e., publications in print and online media, or published online only) published in the U.S. and in English, excluding radio or television transcripts, blogs, and press releases.

**Fig. 1:**
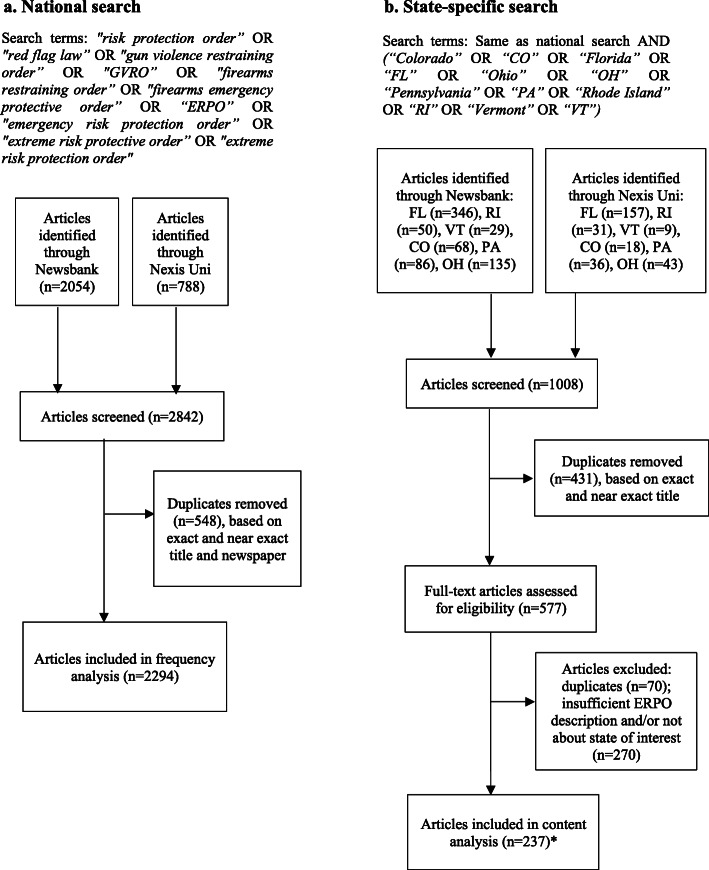
Search strategy for national and state-specific ERPO-related news media coverage, 2018. Asterisk denotes count discrepancy (seven articles were counted twice because they were relevant for two states, yielding a denominator for analyses of 244 articles)

We assembled separate samples of articles for frequency and content analyses (Fig. 1). To plot the frequency of national news coverage from January 1 to December 31, 2018, we retrospectively identified news articles published in 2018 that contained at least one of the following search terms often used to refer to ERPO-like policies: “risk protection order,” “red flag law,” “gun violence restraining order,” “GVRO,” “firearms restraining order,” “firearms emergency protective order,” “ERPO,” “emergency risk protection order,” “extreme risk protective order,” “extreme risk protection order.” Duplicates within and between the databases were manually removed, but each instance of an article that appeared in multiple news sources was counted.

For content analysis, we composed a dataset by searching for all articles related to ERPOs in the six states where ERPO legislation was introduced for the first time after Parkland and subsequently considered by the legislature: Florida, Rhode Island, Vermont, Colorado, Ohio, and Pennsylvania. We included the same search terms used for frequency analysis plus the six states’ names and abbreviations; articles could be published in any U.S. newspaper. For each state, searches spanned from February 15, 2018 (the day after Parkland) until the date of either 1) legislation passage, 2) legislation failure, or 3) the end of the state’s 2018 legislative session (in states where the legislation neither passed nor failed) (Additional file 1, Table S1).

When the same article appeared in multiple newspapers, only the most recent (and if equally recent, the longest) version was coded. After removing duplicates, the three authors who served as coders (AJA, RP, NKW) reviewed full-text articles to ensure they substantively covered ERPO legislation (i.e., included at least a definition or description of an ERPO policy) in at least one of the six states of interest. Articles that contained only the name of the policy and state were not considered sufficient for content analysis. All articles deemed irrelevant or insufficient by a first coder were assessed for relevance by another coder, and all disagreements were adjudicated by the coding team.

### Measures for content analysis

We first developed an a priori codebook based on previous research, expert recommendations, and salient concepts relating to gun violence prevention, media framing, and ERPOs. We iteratively revised the codebook throughout the coding process, adding emergent codes for relevant elements that appeared in the data. The final coding instrument (Additional file 1, Table S2) contained four categories and nine subcategories.

#### Category 1: article descriptives

For each article, we identified the type of author (i.e., journalist, politician, community member), type of article (i.e., news, opinion, letters to editor), and scope of the news outlet (i.e., national or local). Articles published in *The New York Times, The Washington Post, Chicago Tribune, Los Angeles Times, The Wall Street Journal,* and *USA Today* or written for the Associated Press were considered national in scope; all others were considered local. We also classified each article as in favor of, against, or neutral towards ERPOs and confirmed which of the six states the article was about.

#### Category 2: language

##### Name of policy

We searched articles for official names and a common colloquial term (“red flag” laws) used to refer to ERPO-like policies. Gun violence prevention advocacy groups have discouraged use of the name “red flag” order, which, they argue, may be perceived as a subjective process based on “gut feeling” or a policy that targets people with mental illness, thus perpetuating stigma [15, 16]. Instead, they recommend more specific, less stigmatizing language that describes the law’s targeted, data-driven approach. Thus, we classified ERPO policy names appearing in news articles into two groups: those containing the phrase “red flag” (e.g., red flag law, red flag order) and official policy names, which included all other names and their acronyms (e.g., gun violence restraining order, emergency protection order, ERPO).

##### Removal language

Previous research suggests that language plays a role in openness to firearm policy and violence prevention interventions, including for firearm owners [10, 17, 18]. For example, those who may support a policy allowing for temporary firearm removal when someone is in crisis may be dissuaded if language implying permanent prohibition or infringement of rights is used to describe it. Thus, we identified the precise language used to describe the ERPO process of removing access to firearms and classified such language according to frequently-used verbs, such as “seize,” “take away,” “prevent,” and “prohibit.”

##### Key terms

Because small wording differences can influence messaging and impact the overall framing of issues, key terms and phrases used to discuss firearm policy and firearm violence prevention may affect readers’ openness to policy and interventions [10, 16]. Prior research suggests that using words that align with politically conservative and/or gun owners' core values in discussions of gun policy may garner increased policy support [10]. We identified key terms that commonly appear in the public dialogue on gun violence and policy in America (e.g., “gun control,” “common sense,” “Second Amendment”) and on ERPOs in particular (e.g., “warning signs,” “bipartisan,” “imminent threat”) because of their potential to shape the public’s perceptions of and support for such policies.

#### Category 3: contextual information

##### Events

We measured whether articles mentioned three recent, high-profile public mass shootings (occurring in Parkland, FL; Las Vegas, NV; and Newtown, CT), since these events have sparked firearm policy debates and proposals, including ERPO legislation. We also measured whether articles mentioned other specific incidents of firearm violence, including other high-profile shootings and local cases, as well as advocacy events.

##### Case details

Some victim advocates and criminologists have urged the media to avoid publishing the names of perpetrators of violence and unnecessary details of their crimes, which may bring notoriety and encourage copy-cat crimes [19–21]. We identified whether articles mentioned a perpetrator of violence by name, mentioned the race of a perpetrator or victim(s) of violence, or included information about the firearm(s) used in these events (e.g., the type of firearm, how they were acquired, accessories used). We also measured whether articles explicitly stated that an incident was prevented or could have been prevented by using an ERPO.

##### Programs and policies

To better understand the policy context in which ERPOs are discussed, we identified other firearm policies and violence prevention strategies that were mentioned in articles, including background checks, bump stock bans, and assault weapon restrictions. We also measured whether articles mentioned ERPO laws in effect or under consideration in other states or at the federal level.

#### Category 4: anecdotal and research evidence

##### Stakeholders

To see whose perspectives are included and omitted in published media about ERPOs, we identified various stakeholders mentioned or quoted, including politicians or officials, gun violence prevention advocacy groups (e.g., Everytown for Gun Safety) or representatives/members of such groups (including student advocates), firearm industry groups (e.g., National Rifle Association [NRA]) or representatives/members of such groups (including gun rights activists), law enforcement, scientists or researchers, teachers or educators, and health or mental health professionals.

##### Uses of ERPOs

While typically passed in the wake of mass shootings or other high-profile incidents, ERPOs may also be used to prevent other types of firearm-related harm. Research on analagous risk warrant laws in Indiana and Connecticut suggests that these policies are effective tools for suicide prevention [22–24], and emerging data from other states suggests that a majority of ERPOs sought are for cases of risk of harm to self or harm to self and others [25]. Emerging evidence indicates, and experts agree, that ERPOs may be used to remove firearms when someone with cognitive impairment or dementia poses an extreme risk [26, 27]. The media’s communication on the types of risk ERPOs could be used to ameliorate may affect public perceptions as well as the policy’s adoption and use. We measured whether articles explicitly mentioned that ERPOs could be used, or have been used, to prevent mass shootings, suicide, violence among people with cognitive impairments, violence among people with mental illness, domestic violence, community violence, or homicide.

##### Evidence

A public health approach to preventing gun violence is one that is evidence-informed and data-driven. Limited public understanding of the burden of firearm injury and approaches to prevention, however, may affect perceptions of or support for policy [9]. To gauge the frequency of media citations of research evidence, we noted whether articles cited any research or scientific evidence related to firearm violence, including evidence on the burden of gun violence and on the effectiveness or implementation of ERPOs. We also measured whether articles mentioned a need for or lack of research on gun violence.

### Data analysis

For frequency analysis, we calculated the number of articles published per week from January through December 2018 and plotted these frequencies in Excel (Version 16.41) in relation to the introduction and passage of states’ ERPO legislation and public mass shootings. Public mass shootings were identified using the Mother Jones Mass Shootings Database (Additional file 1, Table S3), which defines a mass shooting as a single attack in a public place in which three or more victims are killed [28]. Legislative events were ascertained from *The Trace* and confirmed via state legislature websites (Additional file 1, Table S4) [29].

For content analysis, we uploaded and coded articles in Dedoose (Version 8.2.14). Three of the authors (RP, AJA, NKW) independently coded a subset of ten articles to practice applying the a priori codebook, clarify coding definitions, and add emergent codes. Then, the coding team blindly double-coded a random sample consisting of 20% of the total body of included articles and met regularly to establish consistency, completeness of the codebook, and contextual authenticity. Coding discrepancies were discussed and resolved among the coding team and, when necessary, with a fourth author (CEK). After double-coding 20% of articles, we reached consensus, allowing for single-coding the remaining articles. Coders maintained consistent contact throughout the single-coding phase, including via electronic memos and biweekly meetings, in which further coding clarifications were addressed.

With the exception of article descriptives, all codes were measured dichotomously, indicating the presence or absence of specific content (i.e., words, themes, concepts related to ERPOs) in an article. We calculated the percentage of all content-coded articles (*n* = 244) that contained each item and included qualitative excerpts to illustrate the use of codes in context.

## Results

### Frequency analysis

**Fig. 2:**
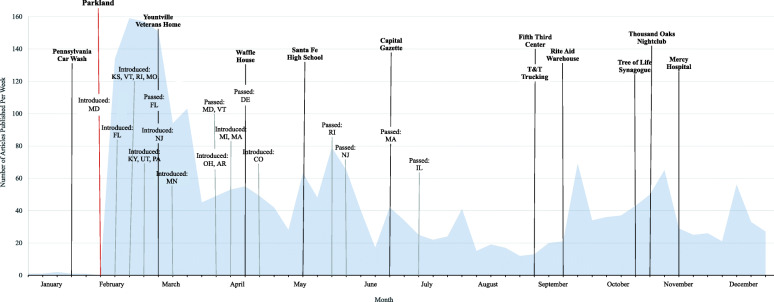
Number of ERPO-related articles published in U.S. newspapers per week, 2018. Each tick mark on the x-axis represents a week, starting with the week of January 4, 2018. Public mass shooting events are noted in boldface. The definition of mass shooting for events noted here is a single attack in a public place in which three or more victims are killed. Public mass shooting events come from Follman et al., 2020 [28] and are available in Additional file 1, Table S3. ERPO policy introductions and passages are noted in non-boldface. Legislative dates were ascertained from state legislature websites and from Campbell et al., 2020 [29] and are available in Additional file 1, Table S4. To view raw data (weekly counts of ERPO-related articles for 2018), see Additional file 1, Table S5

Our search yielded 2294 news articles published in 2018 in U.S. newspapers that mentioned ERPOs. The frequency of such publications increased sharply following Parkland; seven articles mentioning ERPO laws were published in the six weeks prior to the incident, and 798 articles appeared in the six weeks afterwards (Fig. 2). Frequency also increased in relation to subsequent ERPO-related events, including policy proposals and public mass shootings, and remained above the pre-Parkland level throughout 2018.

### Content analysis

#### Category 1: article descriptives

The six-state content analysis sample included 244 articles. The majority (*n* = 186, 76.2%) were written by journalists (Additional file 1, Table S6). Three-quarters (*n* = 189, 77.5%) were news items and the remainder (*n* = 55, 22.5%) were opinion pieces or letters to the editor. Fifteen percent of articles (*n* = 37) appeared in national outlets. Overall, 78.7% (*n* = 192) of articles took a neutral position on ERPOs, and 20.9% (*n* = 51) were favorable towards ERPOs. The greatest shares of articles were about ERPOs in Florida (*n* = 71, 29.1%) and Ohio (*n* = 70, 28.7%).

#### Category 2: language

Just over one-third of articles (*n* = 88, 36.1%) exclusively referred to ERPOs using the term “red flag,” and less than one-third (*n* = 74, 30.3%) exclusively used official policy names (Table 1). The remainder (*n* = 82, 33.6%) used both types of names.

**Table 1:** Language used in a sample of ERPO-related news media (*n* = 244)

	Count	Percent
**Name of policy**		
Any “red flag” name	170	69.7%
"Red flag” name only	88	36.1%
Any official name	156	63.9%
Official name only	74	30.3%
"Red flag” and official name	82	33.6%
**Removal language**		
Seize	73	29.9%
Seize only	28	11.5%
Take away	89	36.5%
Remove	70	28.7%
Prevent	23	9.4%
Prevent only	12	4.9%
Disarm	21	8.6%
Confiscate	27	11.1%
Strip	17	7.0%
Restrict access	16	6.6%
Bar/prohibit/ban/forbid/block	28	11.5%
Keep guns away/separate	14	5.7%
Surrender	5	2.0%
Other	19	7.8%
**Key terms**		
"balance”; “balanced”	10	4.1%
"bipartisan”	30	12.3%
"common ground”; “consensus”	14	5.7%
"common sense”; “sensible”	61	25.0%
"due process”	56	23.0%
"gun control”	79	32.4%
"imminent threat”	21	8.6%
"individual rights”; “constitutional rights”	35	14.3%
"law-abiding”; “responsible gun owners”	23	9.4%
"owe it to victims”	3	1.2%
"politically impractical”	1	0.4%
"Second Amendment”	65	26.6%
"warnings signs”; “red flags”	71	29.1%

Language used to describe the process of removing guns from ERPO respondents ranged from passive and preventive to active and prohibitory. The most common removal language included the verbs “take away” (*n* = 89, 36.5%), “seize” (*n* = 73, 29.9%), and “remove” (n = 70, 28.7%) (Table 1). Prohibitory language, such as “bar someone from owning,” “prohibit firearm possession,” and “ban on buying or possessing,” was used to describe ERPOs in 11.5% (*n* = 28) of articles. The verb “prevent,” with respect to access to firearms, was used in 9.4% (*n* = 23) of articles.

The key terms that appeared most often were “gun control” (*n* = 79, 32.4%), “warning signs; red flags” (*n* = 71, 29.1%), and “Second Amendment” (*n* = 65, 26.6%) (Table 1). Other oft-used key terms included “due process” (*n* = 56, 23.0%), “individual rights; constitutional rights” (*n* = 35, 14.3%), and “bipartisan” (*n* = 30, 12.3%).

The following excerpts demonstrate how these terms can be used to frame ERPOs in different ways. For example, the Editorial Board of the *Daytona Beach News-Journal* (FL), wrote in support of ERPOs (emphasis added):

Called a ‘red flag’ law (also known in its various other forms as ‘gun violence restraining order’ or ‘extreme risk protection order’), it allows police to ask judges to temporarily seize firearms from people who show *warning signs* of violence—as the Parkland shooter had. The goal is to *balance* public safety with *personal rights* by protecting the *Second Amendment* right to own firearms and the Fifth Amendment’s right to *due process* … It’s a targeted solution, identifying individual behavior, not a broad response like outlawing an entire class of firearms that affects even *responsible, law-abiding gun owners* [30].

These terms were also used to reflect concerns about ERPO legislation, as in a piece from *Parsons Sun* (KS) (emphasis added):

Some opponents say even temporary confiscation of a gun before the firearm owner has a chance to challenge the action tramples the *Second Amendment .*.. He argues that by taking the weapons away first and only then letting a gun owner contest that action later, such laws strip people of their *constitutional rights* by denying them a chance to fight in court to keep their weapons [31].

#### Category 3: contextual information

Roughly three-quarters (*n* = 180, 73.8%) of articles mentioned the Parkland shooting, 20.9% (*n* = 51) mentioned the mass shooting at the Mandalay Bay Hotel in Las Vegas, NV and 14.3% (*n* = 35) mentioned the school shooting at Sandy Hook Elementary in Newtown, CT (Table 2).

**Table 2:** Contextual information included in a sample of ERPO-related news media (*n* = 244)

	Count	Percent
**Events**		
Parkland	180	73.8%
Sandy Hook	35	14.3%
Las Vegas	51	20.9%
Other high-profile shooting	68	27.9%
Lesser-known incidents of gun violence	33	13.5%
March for Our Lives, other advocacy event	32	13.1%
**Case details**		
Perpetrator name	64	26.2%
Victim mentioned	35	14.3%
Race of perpetrator or victim	0	–
Characteristics of firearms used in a specific case	48	19.7%
Event was prevented or could have been prevented by an ERPO	32	13.1%
**Programs or policies**		
Any firearm or violence prevention program/policy	106	43.4%
Firearm-related		
Assault weapon restrictions	76	31.1%
Bump stock ban	81	33.2%
High-capacity magazine restrictions	47	19.3%
Other firearm-related	11	4.5%
Prohibiting criteria		
Age limits	63	25.8%
Background checks	104	42.6%
Domestic violence-related	66	27.0%
Other prohibiting criteria	28	11.5%
School security-related	66	27.0%
ERPOs		
Federal or other states’ ERPOs	115	47.1%
Other		
Other firearm law	85	34.8%
Other violence prevention strategy	49	20.1%

More than one-fourth (*n* = 64, 26.2%) mentioned at least one perpetrator of violence by name, and 14.3% (*n* = 35) mentioned at least one victim (Table 2). In the vast majority of cases, the perpetrators mentioned were the gunmen in high-profile mass shootings, including those in Parkland, Las Vegas, Newtown, and others. More than 90% (*n* = 58) of articles that named a perpetrator included mention of the Parkland perpetrator. No article reported the race of a victim or perpetrator of violence.

Approximately one in five (*n* = 48, 19.7%) articles included information about the firearm(s) used in violent events, including the type of firearm, how it was acquired, and any accessories used (Table 2); most of these articles referenced the firearms owned and used by the Parkland shooter. In 13.1% (*n* = 32) of articles, it was explicitly stated that a violent event was prevented or could have been prevented by an ERPO; these statements were also overwhelmingly made in reference to the Parkland shooting. For example, a letter to the editor of *The Plain Dealer* (OH) stated: “Had a red flag bill existed in Florida prior to Feb. 14, America might not be continuing to mourn the senseless deaths of 17 students and teachers” [32].

Overall, 43.4% (*n* = 106) of articles mentioned other firearm or violence prevention policies or interventions (Table 2). These approaches were usually presented as parallel violence prevention solutions rather than as alternatives to ERPO policies. They included policies limiting the availability of certain firearms or accessories, policies based on criteria that prohibit certain individuals from owning or buying firearms (e.g., age limits, background checks, domestic violence perpetration), school security measures, other firearm laws, and other violence prevention strategies (e.g., mental health screening and treatment). Three articles (1.2%) specifically mentioned addressing the “root causes” of gun violence as a prevention strategy.

#### Category 4: anecdotal and research evidence

Officials/politicians were by far the most commonly quoted or mentioned type of stakeholder, appearing in nearly 80% (*n* = 194) of articles (Table 3). Gun violence prevention advocacy groups and student advocates appeared about as frequently as firearm industry groups and gun-rights advocates: in 34.8% (*n* = 85) and 38.1% (*n* = 93) of articles, respectively. Less common were mentions of or quotes by law enforcement (*n* = 27, 11.1%), researchers (*n* = 13, 5.3%), and health and mental health professionals (*n* = 8, 3.3%).

**Table 3:** Anecdotal and research evidence presented in a sample of ERPO-related news media (*n* = 244)

	Count	Percent
**Stakeholders**		
Advocacy groups (incl. student advocates)	85	34.8%
Educators/teachers	5	2.0%
Health professional, mental health professional	8	3.3%
Firearm industry groups (e.g., NRA)	93	38.1%
Law enforcement	27	11.1%
Officials/politicians	194	79.5%
Perpetrator family/representative	7	2.9%
President Donald Trump	20	8.2%
Scientist/researcher	13	5.3%
The community/public as a whole	6	2.5%
**Uses of ERPOs**		
Mass shootings	30	12.3%
Suicide	38	15.6%
Mental illness	12	4.9%
Other	12	4.9%
**Evidence**		
Any type of evidence	62	25.4%
Burden of gun violence	28	11.5%
Evidence on EPROs	29	11.9%
Need for/lack of research	9	3.7%

Articles often described ERPOs as a tool for preventing firearm-related harm, but rarely mentioned their specific uses, including for preventing suicide (mentioned in 15.6% of articles) and preventing mass shootings (mentioned in 12.3% of articles) (Table 3). The following excerpt from an article in *the New York Times* illustrates how the various uses can be conveyed:

[ERPOs] were also used in situations far different from the mass shooting scenarios they were originally conceived to prevent. Most often, guns were removed from people not seen as threats to large groups or public gatherings, but as risks to themselves or to their families, or suffering from debilitating illnesses such as Alzheimer's or alcoholism [33].

One-quarter (*n* = 62, 25.4%) of articles cited any type of scientific evidence related to firearm violence (Table 3). Approximately 12% included data on the burden of gun violence (*n* = 28, 11.5%) and on the implementation or effectiveness of extreme risk laws (*n* = 29, 11.9%), respectively. Articles often referenced the same few research studies or pieces of evidence, including a 2017 study of Connecticut’s risk-warrant law which estimated that one suicide was averted for every 10 to 20 gun seizures [22] and an Everytown for Gun Safety report, which found that 42% of mass shooters exhibited warnings signs or concerning behaviors before committing their crimes [34]. Nine articles (3.7%) mentioned a need for additional gun violence research, and particularly for lifting federal restrictions on gun violence research at the Centers for Disease Control and Prevention.

## Discussion

Media messaging on policy solutions to firearm violence is part of an ongoing national dialogue that shapes and is shaped by public sentiment [9, 10, 14, 35, 36]. Our findings on ERPO coverage underscore past increases in the volume of media coverage about firearm violence in the aftermath of public mass shooting incidents, which function as so-called news hooks, despite accounting for a relatively small share (estimated to be less than 1%) of firearm deaths annually [1, 28].

More than one-third of news articles mentioning ERPOs in 2018 occurred in the six weeks following the February mass shooting at Marjory Stoneman Douglas High School in Parkland, FL, perhaps in part because the shooting was widely portrayed as preventable had an ERPO been available [6]. Smaller increases in ERPO coverage were observed following other high-profile public mass shootings that year. Overall, nearly three in four articles in our six-state content analysis mentioned Parkland.

While other research has also found increases in media attention to gun violence immediately after a mass shooting, often lasting one to two weeks with a subsequent drop to pre-incident levels [14, 35, 37], we found that coverage of ERPOs following the Parkland shooting declined slowly and remained above pre-incident levels throughout the year, signaling a longer-term shift in media attention. This may be a result of the proliferation of ERPO legislation around the country, as well as the high-profile advocacy efforts of students and gun violence prevention groups following Parkland, whose frustrations with political inaction were frequently expressed or cited in the media [38].

The considerable media attention to ERPO policy as a possible preventive measure represents a notable counterpoint to conventional (and more pessimistic) gun violence coverage in the news. Past research has found that news about firearm violence tends to be problem-focused rather than solutions-oriented, despite recommendations that journalists report on possible solutions [35, 37]. For example, one study found that less than 10% of gun violence news articles included any kind of language that evoked optimism about the possibility of ending or reducing gun violence, and when prevention strategies did appear, they were rarely mentioned in reference to specific policy approaches [35].

In contrast, we found that coverage of ERPOs appeared to facilitate discussion of various strategies for preventing gun violence, with more than 40% of ERPO articles mentioning other types of firearm or violence prevention actions, initiatives, or policies. While such solutions-oriented media may reflect newsworthy legislative actions rather than broader shifts in how the news framesgun violence, it nevertheless aligns with recommendations by a coalition of advocacy organizations to highlight the potential for preventive action in messaging on ERPO laws [39]. Consistent with research on gun violence media coverage more broadly, however, only a few ERPO-related articles made reference to addressing structural inequities at the root of firearm violence.

Despite the solutions-oriented nature of ERPO coverage in general, we found discussion about the intended use of ERPOs to reduce or prevent specific types of firearm violence uncommon. When this information did appear, ERPOs for preventing mass shootings were most often mentioned (15% of articles), closely followed by preventing firearm suicide (12% of articles). This highlights a strategic opportunity in the wake of mass shootings to shift the narrative to ensure that the impacts of other types of day-to-day firearm violence, which are more common and make up a much larger share of firearm injuries and deaths, are not ignored, particularly in light of evidence suggesting that ERPO-type laws are particularly effective at preventing firearm suicide [22–24].

Contrary to expert recommendations and the Associated Press Stylebook, one in four ERPO articles mentioned perpetrators of gun violence by name, particularly the Parkland shooter, and one in five described the specific firearms used; only 14% of articles mentioned victims of violence in any detail. News media organizations, victim advocates, criminologists, government officials, and others have urged the media to avoid publishing names and images of perpetrators, especially perpetrators of mass violence, who may be motivated by the prospect of fame and notoriety [19–21]. Journalists and other authors may have room for improvement in this practice. Moreover, despite the common use of the names of perpetrators of mass violence, mentions of their race—mostly white—never appeared. This is notable when compared to media coverage of mass shootings in which the shooter is not white [40–44], as well as interpersonal community violence, which frequently and explicitly attaches Black race to impacted individuals, especially those who have caused harm [35, 36].

References to scientific evidence appeared in just 25% of ERPO articles, and researchers were mentioned or quoted in only 5% of articles. Law enforcement representatives, in contrast, were mentioned or quoted approximately twice as often as researchers, and elected officials appeared in the vast majority (80%) of articles. The relative lack of references to research in EPRO coverage may result from the general paucity of scientific evidence on firearm violence compared to other leading causes of death due to federal government limitations on funding for gun violence research, as well as the challenges in building an evidence base for prevention-oriented policies. Nevertheless, references to scientific evidence on the use and effectiveness of policies in media coverage, even for a nascent policy such as the ERPO, may shape the public narrative and policy support. Additionally, such references can provide an important complement to the prominence often given to law enforcement perspectives on gun violence, particularly given mounting calls for data-informed decision- and policy-making focused not only on intervention and suppression, but also public health approaches that emphasize root causes (e.g., poverty and structural racism) and prevention [45, 46]. Expanding coverage of gun violence to include sections of the news outlet other than crime (e.g., the health care, business, or education beats) may be an important contributor to such a shift.

Increasing attention to ERPOs has also facilitated growing consensus on the importance of the policy name and language used in building public support and political momentum. For example, public opinion polling leading up to a vote on an extreme risk law in Washington State—the only state thus far to pass ERPO policy via ballot measure—found that the names “emergency risk protection order,” “family protection order,” and “proven threat order” were rated most favorably by voters [39]. The name “extreme risk protection order” has since been recommended for widespread use by a coalition of gun violence prevention advocacy organizations because it “describe[s] the purpose of the law in common language and invoke[s] urgency to reflect the situations wherein the law would be used” [39]. The term “red flag law,” in contrast, has been criticized as overly vague and stigmatizing to individuals with mental illness by gun violence prevention experts [15]. Nonetheless, the name “red flag law” appeared in roughly two-thirds of our articles and was the only term used to describe ERPO policy in more than one-third.

To respond to those with concerns about ERPO policy, advocates have also recommended supplementing EPRO messaging with reassuring concepts, such as due process protections [39]. Emerging research suggests that the most acceptable messaging for temporary firearm transfer is that which highlights the temporary nature of many crises and emphasizes that such transfers do not, in themselves, affect an individual’s long-term ability to own firearms [17]. However, coverage of ERPOs more often used prohibitory language, such as “bar someone from owning,” “prohibit firearm possession,” and “ban on buying or possessing,” to describe the implementation of the law rather than preventive language relating to short-term risk, such as “prevent purchases by people deemed to be at risk.”

One-third of articles in our sample used the term “gun control,” a term that may alienate those who see firearm policy as restrictive of Second Amendment rights. For comparison, however, research on media about background checks after the 2012 mass shooting in Newtown, CT found that two-thirds of a sample of articles used the term “gun control” in discussing universal background checks [10]. The relatively lower frequency in ERPO-related media may indicate that ERPOs are not as often perceived as “gun control” relative to other firearm-related policies, or that journalistic practice has changed to reduce use of the term when referring to firearm policy and firearm injury prevention, as experts have recommended [16].

### Limitations

Our approach has several limitations. First, our study is purely descriptive and these results do not allow for claims of causality, such as whether or how elements of media coverage affect passage of legislation. Furthermore, the extent to which the articles we included influenced public perceptions compared with the extent to which public perceptions influenced what was published in the media cannot be determined.

Second, our sample does not represent all media on ERPOs. We searched for news articles in two large databases and excluded non-print news media (e.g., social media, television, and radio), which also influence public dialogue and perceptions. Our searches also had temporal limitations, as we only included media published in the context of a mass shooting and, for content analysis, while legislation was under consideration in states of interest. Articles were only included in our content analysis if they contained at least a basic description of ERPOs, resulting in a sample largely focused on policy and legislation. Additionally, we included opinion and editorial pieces in our content analysis in order to most fully capture the nature of published content on ERPOs. Future work might include only articles authored by journalists or listed as news pieces in order to focus more strictly on how journalists cover ERPOs.

Third, we used binary codes to identify the presence or absence of concepts, which may have resulted in loss of detail. For example, some articles we analyzed included multiple pieces of evidence or quoted numerous politicians. Additionally, some of our findings are de-contextualized. For example, the use of the term “Second Amendment” could reflect a concern about ERPOs (e.g., they “[trample] the Second Amendment”) or a strength of the policy (e.g., they “[protect] the Second Amendment”). Future analysis will further examine the qualitative content of these articles and identify emergent themes.

## Conclusions

As uptake and implementation of extreme risk laws continue in 19 states, and additional states consider these laws, this study provides insights for understanding and reshaping public messaging about ERPOs and firearm violence more generally. Coverage of ERPOs as a means for preventing firearm-related harm may help counter the conventional narrative about gun violence as extreme and unchangeable. Ensuring this framing extends beyond public mass shootings to more common, yet comparatively under-covered, forms of firearm-related harm can help policymakers and the public see, understand, and discuss the broader context in which firearm deaths happen and how they can be prevented. Building the empirical and experiential evidence base on firearm violence prevention, and encouraging use of such evidence in news coverage, may facilitate these more nuanced discussions in the media and beyond.

## Supplementary Information

**Additional file 1: Table S1.** Search periods for six states in ERPO-related news media content analysis. Describes search periods for gathering state-specific samples of print news media using Nexis Uni and Newsbank (“Accesss World News” database) for content analysis. **Table S2.** Codebook for state-specific ERPO-related news media content analysis. Details codebook used to identify contents and common elements of news media on ERPOs. **Table S3.** Public mass shootings in 2018. Lists public mass shootings in 2018, defining mass shooting as a single attack in a public place in which three or more victims are killed. These events are shown in Fig. 2. These dates and public mass shooting details come from Follman et al., 2020 [28]. **Table S4.** Introduction and passage of state ERPO policies, 2018. Lists dates in 2018 on which ERPO legislation was introduced and passed by state. Dates were ascertained from state legislature websites and from Campbell et al., 2020 [29]. **Table S5.** Number of ERPO-related articles published in U.S. newspapers per week, 2018. Provides weekly counts of ERPO-related articles published in U.S. newspapers in the year 2018. These counts are shown in Fig. 2. **Table S6.** Article descriptives for a sample of ERPO-related news media (*n* = 244). Describes author type, article scope (local or national), article type (news or opinion/letters), article proclivity towards ERPOs, and states of interest mentioned.

## Data Availability

The data analysed during the current study are available from the Nexis Uni (via Lexis Nexis online) and Newsbank Access World News news databases using the search terms and time periods specified in the Methods section.

## Abbreviations

U.S: United States
ERPO: Extreme Risk Protection Order
