# Supplementary material for: News media coverage of extreme risk protection order policies surrounding the Parkland shooting: a mixed-methods analysis
Source: BMC Public Health. 2021 Nov 2;21:1986. doi: 10.1186/s12889-021-11909-z (PMC8565081; doi:10.1186/s12889-021-11909-z)
Supplement: Supplementary file 1 — Additional file 1: Table S1. Search periods for six states in ERPO-related news media content analysis. Describes search periods for gathering state-specific samples of print news media using Nexis Uni and Newsbank (“Accesss World News” database) for content analysis. Table S2. Codebook for state-specific ERPO-related news media content analysis. Details codebook used to identify contents and common elements of news media on ERPOs. Table S3. Public mass shootings in 2018. Lists public mass shootings in 2018, defining mass shooting as a single attack in a public place in which three or more victims are killed. These events are shown in Fig. 2. These dates and public mass shooting details come from Follman et al., 2020 [28]. Table S4. Introduction and passage of state ERPO policies, 2018. Lists dates in 2018 on which ERPO legislation was introduced and passed by state. Dates were ascertained from state legislature websites and from Campbell et al., 2020 [29]. Table S5. Number of ERPO-related articles published in U.S. newspapers per week, 2018. Provides weekly counts of ERPO-related articles published in U.S. newspapers in the year 2018. These counts are shown in Fig. 2. Table S6. Article descriptives for a sample of ERPO-related news media (n = 244). Describes author type, article scope (local or national), article type (news or opinion/letters), article proclivity towards ERPOs, and states of interest mentioned. [file 12889_2021_11909_MOESM1_ESM.docx]

**Additional Material** for “News media coverage of extreme risk protection order policies surrounding the Parkland shooting: a mixed-methods analysis”

**Table S1. Search periods for six states in ERPO-related news media content analysis**

| **State** | **Search end date** | **Reason for end of search** |
| --- | --- | --- |
| Florida | 3/8/18 | ERPO legislation passed |
| Vermont | 4/10/18 | ERPO legislation passed |
| Colorado | 5/7/18 | ERPO legislation postponed indefinitely by state senate |
| Rhode Island | 5/31/18 | ERPO legislation passed |
| Pennsylvania | 9/24/18 | ERPO legislation removed from table |
| Ohio | 12/31/18 | Legislative session ended |

We searched Nexis Uni and Newsbank (“Accesss World News” database) for print news media. The search period for content analyses began on 2/15/18. Legislative dates were ascertained from state legislature websites and from Campbell et al., 2020 (29).

**Table S2. Codebook for state-specific ERPO-related news media content analysis**

| **Category/Subcategory/Code** | **Description** |
| --- | --- |
| **Article descriptives** | |
| Author type |  |
| Journalist |  |
| Editorial board |  |
| Politician |  |
| Advocate | Member of a gun violence prevention advocacy organization (e.g., Secretary of the Board of Directors of CeaseFirePA; member of Moms Demand Action for Gun Sense in America) |
| Community member |  |
| Other | Author holds some other distinctive role (e.g., Reporter for student newspaper; "former speaker of Colorado's House of Representatives and the president and CEO of Mental Health Colorado") |
| Don't know | Author's name is not found in article |
| Article scope | Based on the newspaper in which the article was published |
| National | Published in *The New York Times, The Washington Post, Chicago Tribune, Los Angeles Times, The Wall Street Journal,* or *USA Today,* or written by the Associated Press |
| Local | Published in any newspaper not listed above |
| Article type |  |
| News |  |
| Opinion |  |
| Letters to the Editor |  |
| For or against | Author's general position on ERPOs |
| In favor of ERPOs |  |
| Neutral | Author reports on ERPO policy but does not support or oppose the policy |
| Against ERPOs |  |
| State of interest | The state(s) in which the article references ERPO legislation (e.g., "Florida is considering ERPO legislation"). This is not the location of the newspaper. Mentioning a shooting event that occurred within the state, without further discussion of ERPO policy, is insufficient to be considered "about" that state (e.g., "An ERPO law in Florida could have prevented Parkland" is insufficient) |
| FL | Article talks about ERPOs in relation to Florida |
| RI | Article talks about ERPOs in relation to Rhode Island |
| VT | Article talks about ERPOs in relation to Vermont |
| CO | Article talks about ERPOs in relation to Colorado |
| PA | Article talks about ERPOs in relation to Pennsylvania |
| OH | Article talks about ERPOs in relation to Ohio |
| **Language** | |
| Name of policy | Any colloquial or formal name used to refer to ERPO-type policies, including acronyms (e.g., red flag law; gun violence restraining order; extreme risk protection order; GVRO) |
| Removal language | Specific language used for talking about taking someone's guns or prohibiting them from having guns (e.g., confiscate; recover; take away; seize); also includes prevention language (e.g., "prevent from accessing guns") |
| Key terms | Terms should appear exactly or nearly the same as seen here, not simply implied. Only code when relevant to the discussion on ERPOs. |
| "balance"; "balanced" |  |
| "bipartisan" |  |
| "common ground"; "consensus" |  |
| "common sense"; "sensible" |  |
| "due process" |  |
| "gun control" |  |
| "imminent threat" |  |
| "individual rights"; "constitutional rights" |  |
| "law-abiding"; "responsible gun owners" |  |
| "owe it to victims" |  |
| "politically impractical" |  |
| "Second Amendment" |  |
| "warning signs" or "red flags" | Not to be used when referencing name of ERPO policy (i.e., "red flag law") |
| **Contextual information** | |
| Events mentioned |  |
| Parkland | Mentions mass shooting at Marjory Stoneman Douglas High School in Parkland, FL |
| Sandy Hook | Mentions mass shooting at Sandy Hook Elementary in Newtown, CT |
| Las Vegas | Mentions mass shooting at Mandalay Bay Hotel in Las Vegas, NV |
| Other high-profile shooting | Shooting, or potential shooting, events that gained national attention or sparked ERPO-related legislative action in a state (e.g., movie theater in Aurora, CO; Pulse nightclub in Orlando, FL; threat at Fair Haven Union High School, VT) |
| Lesser-known incidents of gun violence | Lesser-known, usually local, incidents of gun violence including suicide (e.g., "Chardon High School shooting in 2012 that left three students dead"; "Oregon, 2016 — A 31-year-old veteran of the U.S. Navy committed suicide with a firearm.") |
| March for Our Lives, other advocacy event | E.g., "The organization is holding a rally Wednesday at the Capitol demanding votes on gun-related bills, not just talk" |
| Case details |  |
| Perpetrator name | Name of perpetrator (e.g., "19-year-old Nikolas Cruz, the alleged Florida gunman") |
| Victim mentioned | Mentions victim(s), usually by name, including indirect victims such as family and friends (e.g., "Fred Guttenberg, whose 14-year-old daughter, Jaime, was among the 17 killed"; "students who survived the shooting"); student survivors may be coded as victims and student advocates |
| Race | Explicit mention of the race of the perpetrator or victim of violence |
| Characteristics of firearms used in a specific case | Details on the firearms belonging to the perpetrator or plans to acquire firearms in the specific case mentioned, including the number, types, means of acquisition, whether they were recovered (e.g., "using an AR-15-style rifle he bought legally") |
| Event was prevented or could have been prevented by an ERPO | Explicitly states this in the article (e.g., "such a law might have prevented a mass shooting like the one in Parkland, Fla.") |
| Programs and policies mentioned | Any mention of a policy, program, or intervention designed to prevent firearm violence or violence generally |
| Assault weapon restrictions |  |
| Bump stock ban |  |
| High-capacity magazine restrictions |  |
| Age limits | E.g., raising age limit to 21 for all firearms |
| Background checks | E.g., expanding background checks; requiring universal background checks |
| Domestic violence-related | Any policies that limit use of firearms by people who commit domestic violence, including domestic violence restraining orders and prohibitions |
| School security-related | E.g., arming teachers; hardening schools |
| Federal ERPO legislation | Mentions ERPO legislation being considered at federal level |
| Other states' ERPOs | Mentions that ERPO laws exist or are being considered in other states (e.g., "Seven states currently have some type of red flag law: California, Connecticut, Washington, Oregon, Indiana, Florida and Rhode Island") |
| Other firearm laws | Any other firearm law not included in other categories (e.g., ban armor piercing bullets; concealed carry laws; Stand Your Ground laws; waiting periods) |
| Other violence prevention strategies | Any other intervention designed to prevent violence and not specifically firearm-related, including mental health treatment (e.g., mental health screening; anonymous tipline) |
| **Anecdotal and research evidence** | |
| Stakeholders quoted or mentioned | Mention or quote of stakeholder, including organizations, groups, and individuals; only code if in relation to discussion on ERPOs |
| Advocacy groups | E.g., Giffords; Everytown; Moms Demand Action |
| Student advocates | E.g., "Thousands of high school and college students rallied across central Ohio" |
| Educators/teachers | E.g., Superintendent; high school chemistry teacher |
| Health professional, mental health professional | E.g., National Alliance on Mental Illness; "Kristin Mathre, chief operating officer ofthe Suncoast Center, a behavioral health nonprofit" |
| Firearm industry groups (e.g., NRA) | Any gun rights group, business that sells guns, pro-gun people, including pro-gun lobbyists, gun-rights attorney, and 2nd Amendment advocates (e.g., "gun proponents"; "Kim Stolfer of Firearms Owners Against Crime, a gun-rights organization") |
| Law enforcement | E.g., ("Cincinnati Police Chief Eliot Isaac"; "backed by bipartisan House leaders, as well as gun control and law enforcement officials") |
| Officials/politicians | Government officials (e.g., "State Sen. Bill Galvano"; "Gov. Gina Raimondo"); also includes attorneys, prosecutors, lawyers |
| Perpetrator family/representative | E.g., "The shooter's aunt, Yudy Martinez Perez"; "Howard Finkelstein, the Broward County public defender representing Cruz" |
| President Donald Trump |  |
| Scientist/researcher | Use in conjunction with "Evidence" code if citing a specific study (e.g., "Duke University researchers") |
| The community/public as a whole |  |
| Uses of ERPOs | Explicitly mentions specific use of ERPOs or scenario in which ERPOs are appropriate or intended to be used |
| Mass shootings | ERPOs used to prevent mass shootings (e.g., "as a means to prevent mass shootings, especially ones where there were numerous warning signs") |
| Suicide | ERPOS used to prevent suicide (e.g., "Mass shootings grab the headlines, but suicides may also be prevented through red-flag laws") |
| Mental illness | ERPOs used to prevent violence by those with severe mental health disorders (e.g., "a proposed law that could, at least temporarily, keep guns away from people with a mental illness") |
| Cognitive impairment | ERPOs used to prevent violence by those with dementia or other cognitive impairment (e.g., "Most often, guns were removed from people not seen as threats to large groups or public gatherings, but as risks to themselves or to their families, or suffering from debilitating illnesses such as Alzheimer's or alcoholism") |
| Community violence | ERPOs used to prevent community violence (e.g., "an important new tool they can use to protect our communities from gun violence") |
| Domestic violence | ERPOs used to prevent domestic violence (e.g., "it could also be used to prevent suicide or domestic violence") |
| Homicide | ERPOs used to prevent homicide (e.g., "studies show that restricting access to firearms in these circumstances --even temporarily -- reduces the likelihood of suicide or homicide") |
| Evidence | Article cites any data or scientific evidence related to firearm violence (e.g., risk factors; effectiveness of gun laws) |
| Burden of gun violence | Cites data that characterizes the burden of firearm violence (e.g., "Eighty-nine percent of the gun deaths in Vermont from 2011 through 2016 were suicides; this is much higher than the 60 percent of gun deaths nationally that are suicides") |
| Evidence on ERPOs | Presents or discusses the existing evidence for ERPOs, including their implementation and effectiveness (e.g., "In Connecticut, where 762 gun seizure cases were carried out from 1999 to June 2013, a study by researchers at Duke University estimated that the law had averted approximately one suicide for every 10 to 11 gun seizure cases.") |
| Need for/lack of research | Explicitly acknowledges the need for or lack of firearm violence research (e.g., "Among the leading causes of death in America, gun violence is the least-researched. That also makes it the least understood") |

**Table S3. Public mass shootings in 2018**

| **Name** | **Date** | **City and State** | **Number of fatalities** |
| --- | --- | --- | --- |
| Pennsylvania carwash shooting | 1/28/18 | Melcroft, PA | 4 |
| Marjory Stoneman Douglas High School (Parkland) shooting | 2/14/18 | Parkland, FL | 17 |
| Yountville veterans home shooting | 3/9/18 | Yountville, CA | 3 |
| Waffle House shooting | 4/22/18 | Nashville, TN | 4 |
| Santa Fe High School shooting | 5/18/18 | Santa Fe, TX | 10 |
| Capital Gazette shooting | 6/28/18 | Annapolis, MD | 5 |
| Fifth Third Center shooting | 9/6/18 | Cincinnati, OH | 3 |
| T&T Trucking shooting | 9/12/18 | Bakersfield, CA | 5 |
| Rite Aid warehouse shooting | 9/20/18 | Perryman, MD | 3 |
| Tree of Life synagogue shooting | 10/27/18 | Pittsburgh, PA | 11 |
| Thousand Oaks nightclub shooting | 11/7/18 | Thousand Oaks, CA | 12 |
| Mercy Hospital shooting | 11/19/18 | Chicago, IL | 3 |

The definition of mass shooting used is a single attack in a public place in which three or more victims are killed. Data from Follman et al., 2020 (28).

**Table S4. Introduction and Passage of State ERPO Policies, 2018**

| **Event** | **Date** |
| --- | --- |
| Introduced (MD) | 2/9/18 |
| **Introduced (FL)** | 2/21/18 |
| Introduced (KS) | 2/22/18 |
| **Introduced (VT)** | 2/23/18 |
| **Introduced (RI)** | 2/27/18 |
| Introduced (MO) | 2/28/18 |
| Introduced (KY) | 3/1/18 |
| Introduced (UT) | 3/1/18 |
| **Introduced (PA)** | 3/5/18 |
| Introduced (NJ) | 3/8/18 |
| Passed (FL) | 3/9/18 |
| Introduced (MN) | 3/19/18 |
| **Introduced (OH)** | 4/5/18 |
| Passed (MD) | 4/9/18 |
| Introduced (AR) | 4/10/18 |
| Passed (VT) | 4/11/18 |
| Introduced (MI) | 4/12/18 |
| Introduced (MA) | 4/13/18 |
| Passed (DE) | 4/24/18 |
| **Introduced (CO)** | 4/30/18 |
| Passed (RI) | 6/1/18 |
| Passed (NJ) | 6/13/18 |
| Passed (MA) | 7/3/18 |
| Passed (IL) | 7/16/18 |

Legislative dates were ascertained from state legislature websites and from Campbell et al., 2020 (29). The six states in boldface were included in our content analysis; these were the only states in which ERPO legislation was (1) introduced *for the first time* after Parkland and (2) seriously considered by the legislature in the 2018 legislative session.

**Table S5. Number of ERPO-related articles published in US newspapers per week, 2018**

| **Week** | **Count** |
| --- | --- |
| 1/4/2018-1/10/2018 | 1 |
| 1/11/2018-1/17/2018 | 1 |
| 1/18/2018-1/24/2018 | 2 |
| 1/25/2018-1/31/2018 | 1 |
| 2/1/2018-2/7/2018 | 1 |
| 2/8/2018-2/14/2018 | 0 |
| 2/15/2018-2/21/2018 | 134 |
| 2/22/2018-2/28/2018 | 159 |
| 3/1/2018-3/7/2018 | 157 |
| 3/8/2018-3/14/2018 | 151 |
| 3/15/2018-3/21/2018 | 94 |
| 3/22/2018-3/28/2018 | 103 |
| 3/29/2018-4/4/2018 | 45 |
| 4/5/2018-4/11/2018 | 49 |
| 4/12/2018-4/18/2018 | 53 |
| 4/19/2018-4/25/2018 | 55 |
| 4/26/2018-5/2/2018 | 49 |
| 5/3/2018-5/9/2018 | 42 |
| 5/10/2018-5/16/2018 | 28 |
| 5/17/2018-5/23/2018 | 63 |
| 5/24/2018-5/30/2018 | 48 |
| 5/31/2018-6/6/2018 | 79 |
| 6/7/2018-6/13/2018 | 65 |
| 6/14/2018-6/20/2018 | 40 |
| 6/21/2018-6/27/2018 | 17 |
| 6/28/2018-7/4/2018 | 42 |
| 7/5/2018-7/11/2018 | 34 |
| 7/12/2018-7/18/2018 | 25 |
| 7/19/2018-7/25/2018 | 22 |
| 7/26/2018-8/1/2018 | 24 |
| 8/2/2018-8/8/2018 | 41 |
| 8/9/2018-8/15/2018 | 15 |
| 8/16/2018-8/22/2018 | 19 |
| 8/23/2018-8/29/2018 | 17 |
| 8/30/2018-9/5/2018 | 12 |
| 9/6/2018-9/12/2018 | 13 |
| 9/13/2018-9/19/2018 | 20 |
| 9/20/2018-9/26/2018 | 21 |
| 9/27/2018-10/3/2018 | 69 |
| 10/4/2018-10/10/2018 | 34 |
| 10/11/2018-10/17/2018 | 36 |
| 10/18/2018-10/24/2018 | 37 |
| 10/25/2018-10/31/2018 | 43 |
| 11/1/2018-11/7/2018 | 50 |
| 11/8/2018-11/14/2018 | 65 |
| 11/15/2018-11/21/2018 | 29 |
| 11/22/2018-11/28/2018 | 25 |
| 11/29/2018-12/5/2018 | 26 |
| 12/6/2018-12/12/2018 | 21 |
| 12/13/2018-12/19/2018 | 56 |
| 12/20/2018-12/26/2018 | 33 |
| 12/27/2018-12/31/2018 | 27 |

**Table S6. Article descriptives for a sample of ERPO-related news media (n=244)**

|  | Count | Percent |
| --- | --- | --- |
| **Author Type** |  |  |
| Politician | 11 | 4.5% |
| Journalist | 186 | 76.2% |
| Editorial board | 17 | 7.0% |
| Advocates | 2 | 0.8% |
| Community member | 10 | 4.1% |
| Other | 4 | 1.6% |
| Don't know | 14 | 5.7% |
| **Article Scope** |  |  |
| Local | 207 | 84.8% |
| National | 37 | 15.2% |
| **Article Type** |  |  |
| News | 189 | 77.5% |
| Opinion/Letters | 55 | 22.5% |
| **For or Against** |  |  |
| In favor of ERPOs | 51 | 20.9% |
| Neutral | 192 | 78.7% |
| Against ERPOs | 1 | 0.4% |
| **State of Interest** |  |  |
| FL | 71 | 29.1% |
| RI | 36 | 14.8% |
| VT | 17 | 7.0% |
| CO | 13 | 5.3% |
| PA | 37 | 15.2% |
| OH | 70 | 28.7% |
